# Gender and Use of Substance Abuse Treatment Services

**Published:** 2006

**Authors:** Carla A. Green

**Affiliations:** Carla A. Green, Ph.D., MPH, is a senior investigator at the Center for Health Research, Kaiser Permanente Northwest, and a clinical associate professor in the Department of Public Health and Preventive Medicine and the Department of Psychiatry at Oregon Health & Science University, both positions in Portland, Oregon

**Keywords:** health services research, health care delivery research, primary health care, mental health care, AOD (alcohol and other drug) abuse, treatment outcome in HSR (health services research), AODU (AOD use) treatment method, treatment referral, treatment barriers, gender differences, single gender group, mixed gender group, women, female

## Abstract

Women are more likely than men to face multiple barriers to accessing substance abuse treatment and are less likely to seek treatment. Women also tend to seek care in mental health or primary care settings rather than in specialized treatment programs, which may contribute to poorer treatment outcomes. When gender differences in treatment outcomes are reported, however, women tend to fare better than men. Limited research suggests that gender-specific treatment is no more effective than mixed-gender treatment, though certain women may only seek treatment in women-only programs. Future health services research should consider or develop methods for (1) improving care for women who seek help in primary care or mental health settings, (2) increasing the referral of women to specialized addiction treatment, (3) identifying subgroups of women and men who would benefit from gender-specific interventions, and (4) addressing gender-specific risk factors for reduced treatment initiation, continuation, and treatment outcomes.

In the 1970s and 1980s, practitioners and researchers began to call attention to how little was known about providing appropriate care for women with substance abuse problems, particularly alcoholism (Schmidt and Weisner 1995). Research traditionally had focused on how men fared in substance abuse treatment, and treatment programs were ill-equipped to help women. In response, government organizations began to support research and treatment for women, and significant numbers of researchers and practitioners focused on understanding and addressing gender differences in treatment access, treatment provision, and outcomes (Schmidt and Weisner 1995). Researchers began examining the characteristics and social circumstances of women with substance abuse problems, identifying factors that interfered with detecting and diagnosing women who needed help. They also studied the barriers that prevented women from entering treatment and gender-specific issues related to women’s success in treatment. These efforts have resulted in a large body of research addressing gender differences in treatment-seeking, access to care, retention in care, and treatment outcomes.

Over this same period, many treatment programs also began to pay greater attention to the women in their programs and their special needs. Today many (although not all) treatment programs offer gender-specific or gender-sensitive services, such as gender-matching with counselors, mixed-gender treatment groups led by male and female co-leaders, gender-specific treatment groups, and gender-specific treatment content. Many programs also provide ancillary or wraparound services, such as child care and parenting groups, which facilitate women’s treatment entry and continuation. In addition, significant numbers of treatment programs serve women only, target pregnant women or adolescent girls, or offer specialized parenting services for women and their children.

These profound changes in research and treatment programs have occurred at the same time as other important social changes in the United States, such as women’s increased participation in the workforce, greater similarities in men’s and women’s patterns of substance use, and increased public knowledge about substance-abuse-related problems and their treatment. As a result, health services researchers who study gender and substance abuse treatment are finding it difficult to know whether findings from earlier research are still applicable in current settings. Consequently, the following review sometimes specifically distinguishes earlier research findings (i.e., from the 1970s and the 1980s) from more recent research findings.

This article examines gender differences in the prevalence of substance use and related problems, the identification of such problems, treatment-seeking and access, retention in treatment, and treatment outcomes.

## Gender and the Prevalence of Substance Use and Substance-Related Problems

Research on how gender influences substance use and substance-abuse-related problems has established clear differences between women and men in several important areas. Women typically consume less alcohol than men when they drink, drink alcohol less frequently, and are less likely to develop alcohol-related problems than men (Fillmore et al. 1997). Similarly, women are less likely than men to use illicit drugs and to develop drug-related problems (Greenfield et al. 2003*a*).

Conversely, when women *do* develop substance abuse problems, they tend to develop them faster than men do. For example, although women tend to be older than men, on average, when they begin a pattern of regular drunkenness, women’s drinking-related problems (e.g., loss of control over drinking, negative consequences of drinking) appear to progress more quickly than those of men (Randall et al. 1999). This faster progression also means that women experience shorter intervals than men between onset of regular drunkenness and first encountering the negative consequences of drinking, which include physical problems, interpersonal difficulties, negative intrapersonal changes (such as in personality or self-esteem), poor impulse control, and reduced ability to maintain normal social roles and responsibilities. Women also experience shorter intervals between first loss of control over drinking and onset of their most severe drinking-related consequences, and shorter intervals between onset of regular drunkenness and treatment-seeking (Randall et al. 1999). Women report more severe problems and experience more health-related consequences from substance use (Bradley et al. 1998), and their substance-related problems interfere with functioning in more life domains compared with men (Fillmore et al. 1997).

**At a Glance**

**The Nature of Women’s Substance Abuse Problems**

- Women are less likely than men to use illicit drugs and develop drug-related problems (Greenfield et al. 2003*a*).
- Women drinkers tend to drink less alcohol less often than men do and are less likely than men to develop alcohol-related problems (Fillmore et al. 1997).
- When women do develop substance abuse problems, they report problems of greater severity and experience more health-related consequences (Bradley et al. 1995).
- Women’s problems related to substance abuse interfere with functioning in more areas of life than men’s do (Fillmore et al. 1997).
- Women are older than men are when they begin drinking to intoxication, but once they develop a pattern of regular intoxication, they:
  – Encounter drinking-related problems more quickly than men (Randall et al. 1999)
  – Lose control over their drinking more quickly than men (Randall et al. 1999).
- Recent research shows that women’s and men’s substance use patterns have become more similar in the past few years (McPherson et al. 2004).
- Women make up about one-third of the population with alcohol problems and slightly less than half of those who have problems with other drugs (Greenfield et al. 2003*a*).

Recent narrowing of the differences in men’s and women’s substance use patterns and attitudes (McPherson et al. 2004) raises additional concerns because of women’s greater susceptibility to substance-related problems. Based on recent U.S. prevalence estimates, women make up about one-third of people with alcohol problems and slightly less than half of those who have problems with other drugs (Greenfield et al. 2003*a*).

**At a Glance**

**Barriers to Treatment**

- Women are more likely than men to encounter barriers that prevent them from seeking or following through with treatment (Brady and Ashley 2005).
- Women are more likely to experience economic barriers to treatment (Brady and Ashley 2005).
- Women are more likely to have difficulty attending regular treatment sessions because of family responsibilities (Brady and Ashley 2005; Brady and Randall 1999).
- Providing comprehensive services, such as housing, transportation, education, and income support, reduces post-treatment substance use among both men and women, but greater numbers of women need such services (Marsh et al. 2004, 2000).
- Women are more likely to report feeling shame or embarrassment because they are in substance abuse treatment (Thom 1987).
- Anxiety or depressive disorders, which tend to be more prevalent and severe among women, may prevent women from seeking help with substance abuse problems (Brady and Randall 1999).

## Gender and the Identification of Substance Abuse Problems

A person’s gender has the potential to affect several critical junctures along the pathway to seeking substance abuse treatment. Identification of a problem is the first step toward treatment, whether by the person needing treatment, or by a family member, health care professional, employer, or government agency. The likelihood that a person’s substance abuse problem will be identified appears to differ by gender in some settings. For example, compared with men, substance abuse problems among women, particularly older women (National Center on Addiction and Substance Abuse 1998), are less likely to be identified in health care settings (Brienza and Stein 2002). Women with substance abuse are more likely than men to be identified through contacts with child protective services (Fiorentine et al. 1997; Grella and Joshi 1999). Women also are less likely than men to be referred for substance abuse treatment by their employers or schools (Morgenstern and Bux 2003) and are more likely to have family members, friends, and partners who use drugs and support their substance use (Bendtsen et al. 2002; Grella and Joshi 1999; Center for Substance Abuse Treatment 1994; Kelley et al. 1996; Kline 1996).

## Treatment-Seeking

Once people realize they have a substance abuse problem, they must decide or be convinced that they need help—through personal reflection, feedback from others, or legal, employer, or family mandates. Information about possible differences in how men and women go through these processes is limited. Little is known about how families interact when a family member has substance abuse problems, about how the gender of that person influences how families or employers communicate about or manage these problems (see Room et al. 2004 for an intriguing exception), or about how gender might influence reflection prior to treatment-seeking.

Researchers have determined that employed women seeking treatment for alcohol problems are less likely than men to be married and, if married, are less likely to have had spouses who played a role in referral to treatment (Blum et al. 1995). Men who receive suggestions to cut down or stop drinking are more likely to enter treatment, whereas such suggestions do not appear to predict treatment entry for women (Weisner 1993). Early research suggested that women were discouraged by family members from seeking treatment (Beckman and Amaro 1986), and an older Swedish study (Dahlgren and Myrhed 1977) found that women were more likely than men to enter treatment after serious acute complications of their substance use (e.g., unconsciousness, suicide attempts). Women and men do not appear to differ, however, in their perceptions about the need for treatment (Wu and Ringwalt 2004).

In the context of mandated treatment, sources of mandates differ for men and women (Grella and Joshi 1999), with men more likely to be mandated to treatment by employers, through the criminal justice system, and by their families. Women, in contrast, are more often referred by a social worker, suggesting family service agency involvement in their treatment entry (Grella and Joshi 1999). To date, researchers have not directly addressed how gender affects the processes leading to such mandates.

## Barriers to Treatment

Once people recognize that they have a substance abuse problem and decide to seek treatment, they still must overcome a variety of barriers to finding and accessing treatment resources. Many studies provide evidence for gender differences in the type, strength, and number of barriers people encounter as they consider and attempt to access treatment. For example, Brady and Ashley (2005) reported that women are more likely than men to experience economic barriers when seeking treatment. They also are more likely to have trouble finding the time to attend regular treatment sessions because of family responsibilities and must overcome problems with transportation. Both men and women must overcome the stigma associated with seeking treatment, but women are particularly susceptible to feeling stigmatized (Brady and Ashley 2005).

Lack of information about treatment options––their availability and likelihood of success––is another barrier. Few studies have investigated whether men and women differ significantly in their knowledge in these areas.

Limitations in everyday functioning caused by substance abuse and dependence and common co-occurring conditions, such as mental illness, also can prevent people from accessing treatment. Although both men and women are likely to experience these functional limitations, anxiety and depressive disorders tend to be more prevalent (Hesselbrock and Hesselbrock 1997) and more severe among women with substance abuse problems. For this reason, women may be less likely to seek or follow through with care (Brady and Ashley 2005).

Finally, women from some ethnic groups (such as Hispanic women) may experience cultural barriers (e.g., language problems [U.S. Department of Health and Human Services 2001]) to seeking treatment (Weiss et al. 2003). Moreover, older women are more likely than younger women to encounter physicians who do not believe substance abuse treatment is effective for them, and to have insurance carriers that deny them coverage for treatment (National Center on Addiction and Substance Abuse 1998).

In sum, women are more likely than men to encounter multiple barriers to treatment entry, making them less likely to seek care for their substance-related problems (Brady and Ashley 2005).

**At a Glance**

**Treatment Course and Outcomes**

Because of the characteristics of women with substance abuse problems and the obstacles to treatment they face, many researchers have suggested that women would be less likely to seek, begin, or complete treatment, and would therefore have poorer long-term outcomes (Schmidt and Weisner 1995).

BUT:

- Most recent studies suggest that gender either has no effect on treatment initiation or, if it has an effect, women are more likely than men to initiate treatment (Weisner et al. 2001; Green et al. 2002; Timko et al. 2002).
- Women now appear at least as likely as men to engage in and complete treatment (Brady and Ashley 2005).
- Men and women are equally likely to complete treatment, but women who complete are nine times more likely to be abstinent than women who do not; men who complete treatment were only three times more likely to be abstinent than men who do not (Green et al. 2004).
- Current research suggests that women’s treatment outcomes are as good as, or better than, men’s.
- Women in substance abuse treatment are less likely to relapse than men in treatment. When women relapse, their reasons for relapse differ from men’s:
  – Women are more likely to relapse when their romantic partners are substance users (Rubin et al. 1996).
  – Women are more likely to report personal problems before relapse (McKay et al. 1996).
- Women who have been in treatment have better long-term recovery outcomes than men (Dawson et al. 2005; Weisner et al. 2003).

## Treatment Access

Consistent with these concerns, early research suggested that women with substance abuse problems were less likely to seek help than men with similar problem severity. More recent research suggests that rates of treatment access have improved, with women seeking care at rates similar to those of men, at least in the years following problem onset (Dawson 1996). Similar rates of treatment entry, however, may indicate that women continue to have reduced access compared with men because women consistently use more medical services in other settings than men do (Bertakis et al. 2000). Furthermore, although women’s access appears to have improved generally, some studies continue to find fewer admissions to substance abuse treatment among women (Westermeyer and Boedicker 2000; Arfken et al. 2002).

Various barriers experienced by women, particularly those related to stigma, may influence where women seek help, and whether they seek it from a health professional, a self-help group, or from another source, such as a member of the clergy. Women have been more likely than men to seek help in mental health and primary care settings rather than in substance abuse treatment settings (Weisner and Schmidt 1992). Recent research suggests that care obtained in these nonspecialty settings can lead to poorer treatment outcomes than those achieved at specialty treatment settings. For example, Mojtabai (2005) found that people receiving specialty substance abuse treatment services were less likely to continue substance use than those receiving mental health services. Other recent studies show that, consistent with the greater severity of women’s alcohol-related problems when seeking treatment, women have longer inpatient stays than men, and increasingly are more likely to use self-help programs such as Alcoholics Anonymous (with or without formal treatment) (Timko et al. 2002). Women also are more likely to benefit from these self-help programs than men are (Timko et al. 2002).

In sum, research findings indicate that help-seeking for substance abuse, dependence, and substance-related problems is affected by gender and gender-related characteristics (Weisner and Schmidt 2001). Research is needed to determine the relative value of improving substance abuse treatment services for women in the settings in which they currently seek care (such as in mental health and primary care settings) compared with the value of working to increase referrals to specialty addiction treatment. Improving the latter may be particularly important, given women’s greater needs upon entering treatment: Mental health and primary care settings may be significantly less prepared to manage women’s ancillary service needs and the complexity of their disorders.

## Gender Differences Upon Treatment Entry

Research has found that women seeking treatment for alcohol or other drug problems have more severe problems (Arfken et al. 2001), are younger, have lower education levels (Wechsberg et al. 1998), and have lower incomes (Brady et al. 1993) than men seeking treatment. Women are more likely to have experienced emotional, physical, and sexual abuse (Wechsberg et al. 1998); to have more severe depressive symptoms when depressed (Pettinati et al. 1997); and to be more hostile than men upon treatment entry (Robinson et al. 2001). Women also report more physical and mental health problems (Brady et al. 1993) and greater concerns about child-related issues (Wechsberg et al. 1998) than men do. In addition, women entering treatment for alcohol-related problems are more likely than men to identify factors other than drinking (e.g., stressful life events, mental health symptoms) as their primary problems and, at least as indicated by earlier studies, have been more likely to report shame and embarrassment at treatment entry (Thom 1987). These differences have led many to conclude that women would be less likely to seek, initiate, or complete treatment, and would therefore have poorer long-term outcomes.

## Initiation, Engagement, and Retention in Treatment

Once they have made contact with a source of help, people may or may not proceed to the next step in the process— initiating treatment. They have many choices about initiation, including choice of setting—that is, whether in a formal substance abuse treatment program, another health care setting, within a support group, or with an individual helper. After a recommendation for continuation (often following assessment in a substance abuse treatment setting), people must decide whether to continue along the path they have chosen, seek other services, or decline help. Those who continue then must repeatedly choose whether or not to continue their involvement. This is true even if they have been mandated to treatment. People who select a formal treatment setting must decide whether or not they will complete the treatment program as it has been designed. Research has begun to assess the ways that gender affects the different steps in the treatment process: initiation, engagement and continuation in treatment, completion of the program, and subsequent outcomes.

Most recent studies suggest that gender either has no effect on initiation, or that if it does have an effect, women are more likely than men to initiate treatment (Weisner et al. 2001; Green et al. 2002; Timko et al. 2002). Similarly, research indicates that changes in the provision of care seem to have allayed concerns that women’s continuing engagement in treatment might be hindered by programs’ insensitivity to women’s needs. Women now appear at least as likely as men to engage in and complete treatment, although women from certain subgroups may be at risk for not completing treatment. For example, African American women— as well as women with lower incomes who are unmarried, unemployed, or have psychological problems of greater severity—are less likely to continue with treatment (Mertens and Weisner 2000). Research is needed to identify (1) subgroups at risk for not continuing treatment, (2) modifiable barriers to treatment completion among members of these groups, and (3) appropriate remedies for these barriers.

## Gender and Treatment Outcomes

Many recent research efforts have addressed gender differences in treatment outcomes (which are defined in various ways, including abstinence rates and number of days substances were used in a particular period). Despite concerns that women would fare worse than men, current evidence suggests that, overall, women’s substance abuse treatment outcomes are as good as, or better than, men’s treatment outcomes. For example, one recent study found that men and women were equally likely to complete treatment, but women who completed treatment were nine times more likely to be abstinent than women who did not complete, whereas men who completed treatment were only three times more likely to be abstinent than men who did not complete treatment (Green et al. 2004).

Researchers also have identified many factors that differ by gender and affect treatment outcomes in important ways—including income, education, employment, types of substances used, psychiatric disorders and symptoms, marital status, self-efficacy, history of sexual abuse, and children in the home (Green et al. 2004; Greenfield et al. 2003*b*, 2000, 1998, 2002). This suggests that addressing risks differentially, by gender, may help improve both the treatment process and outcomes for men and women. Recognizing these risks, and the potentially differential needs of men and women, has led to the development of gender-specific treatment programs and the provision of women-centered ancillary services. For example, evidence shows that providing services such as child care helps keep women in treatment (Brady and Ashley 2005).

Research on the benefits of gender-specific treatment is less clear than evidence on the benefits of ancillary services, because few studies have compared gender-specific treatment with mixed-gender treatment (Orwin et al. 2001; Smith and Weisner 2000). One recent study randomly assigned female participants to women-only versus mixed-gender programs and found no differences in outcomes (Kaskutas et al. 2005).

Yet, as is true for other aspects of the treatment process, some subgroups of women may be more likely to benefit from gender-specific treatment. For example, substance-abusing women with post-traumatic stress disorder (PTSD) may benefit significantly more from gender-specific programs designed to address PTSD and addiction problems simultaneously. These programs provide gender-specific content and address, in a comfortable setting (i.e., with only female participants), traumatic experiences and sexual assault (Hien et al. 2004). (Men with PTSD resulting from combat experiences may similarly benefit from male-only groups.) Pregnant and perinatal women also have needs that may be more easily addressed in women-only programs. Although such programs are effective at improving outcomes (Orwin et al. 2001), important pregnancy, labor, delivery, and lactation concerns, as well as other needs (e.g., child care, service coordination, and mental health care) remain unaddressed among these women, particularly among those who also have mental health problems (Grella 1997). Finally, certain women may not seek treatment if women-only treatment programs are not available (Weisner 2005).

Rather than relying solely on gender-specific treatment, some researchers have examined ways to improve treatment by making it gender-sensitive. One approach is to match therapist and client gender or to match therapeutic modality to gender. Results of these efforts have been equivocal. Project MATCH found that matching gender and therapeutic modality had no effect on outcomes (Project MATCH Research Group 1997). Studies addressing therapist–client gender-matching have produced a range of outcomes— with some finding no effects, and others finding greater empathy and therapeutic alliance, longer treatment episodes, and higher rates of abstinence, but also more post-treatment psychiatric symptoms (Fiorentine and Hillhouse 1999; Sterling et al. 1998, 2001; McKay et al. 2003; Nurco et al. 1988). Additional research could help to determine the types of clients best served by same-gender or opposite-gender therapists, and to illuminate the mechanisms by which matching influences treatment process and outcomes.

## Gender and Relapse

Not surprisingly, women who have stopped using substances relapse under different circumstances than men do. For example, women are more likely to relapse in the presence of a romantic partner than men are, and are less likely to relapse when they are alone (Rubin et al. 1996). Women also are more likely to report interpersonal problems before relapse (McKay et al. 1996). Consistent with findings of women’s better outcomes in other domains (e.g., post-treatment abstinence, retention in treatment), women are less likely than men to relapse overall (Rubin et al. 1996), and women tend to have better long-term recovery outcomes (Dawson et al. 2005; Weisner et al. 2003). Such results suggest that future research on gender differences in treatment outcomes should focus on improving the understanding of the underlying factors which differ by gender and predict better outcomes (such as better therapeutic alliances among women in treatment) and reduced relapse. Such a focus might further improve treatment outcomes for both men and women.

## Summary and Conclusions

Over the past two decades, health services researchers have successfully identified gender differences in patterns of substance use, health and social effects of substance use, pathways to treatment for substance abuse problems, and substance abuse treatment processes and outcomes. As a result of the efforts of treatment programs to address women’s needs, and the efforts of researchers to document the effectiveness of treatment for women, it is known that, in general, specialty addiction treatment is at least as effective for women as it is for men.

At the same time, this work has identified both different and common predictors of treatment access and outcomes for men and women. Now it is possible to target gender-specific factors that increase the risks that substance abuse problems will go undetected or lead to reduced treatment initiation and treatment completion. It also is possible to identify factors that could reduce relapse and improve outcomes. For example, because women continue to seek substance abuse treatment in primary care and mental health settings, care providers in these settings could be trained to identify and refer women to specialty addiction services. Conversely, integrated programs could be developed to provide care to women in places other than specialized treatment agencies or departments (e.g., primary care or mental health settings). Similarly, men who have been victims of domestic violence or forced sex might benefit from approaches developed for women with such histories.

Finally, this body of research suggests that a large proportion of men and women do well in mixed-gender treatment settings, and for these people, such settings will likely be more cost-effective than providing gender-specific treatment. However, some individuals or subgroups (female and male) may benefit in important ways from gender-specific treatment. Adequate assessment and appropriate treatment—whether in gender-sensitive mixed-gender programs or gender-specific programs—are critical to improving clinical outcomes for many people who currently are not well served.
